# Soil Type Dependent Rhizosphere Competence and Biocontrol of Two Bacterial Inoculant Strains and Their Effects on the Rhizosphere Microbial Community of Field-Grown Lettuce

**DOI:** 10.1371/journal.pone.0103726

**Published:** 2014-08-06

**Authors:** Susanne Schreiter, Martin Sandmann, Kornelia Smalla, Rita Grosch

**Affiliations:** 1 Julius Kühn-Institut – Federal Research Centre for Cultivated Plants (JKI), Institute for Epidemiology and Pathogen Diagnostics, Braunschweig, Germany; 2 Leibniz Institute of Vegetable and Ornamental Crops Großbeeren/Erfurt e.V., Department Plant Health, Großbeeren, Germany; Universidad de Salamanca, Spain

## Abstract

Rhizosphere competence of bacterial inoculants is assumed to be important for successful biocontrol. Knowledge of factors influencing rhizosphere competence under field conditions is largely lacking. The present study is aimed to unravel the effects of soil types on the rhizosphere competence and biocontrol activity of the two inoculant strains *Pseudomonas jessenii* RU47 and *Serratia plymuthica* 3Re4-18 in field-grown lettuce in soils inoculated with *Rhizoctonia solani* AG1-IB or not. Two independent experiments were carried out in 2011 on an experimental plot system with three soil types sharing the same cropping history and weather conditions for more than 10 years. Rifampicin resistant mutants of the inoculants were used to evaluate their colonization in the rhizosphere of lettuce. The rhizosphere bacterial community structure was analyzed by denaturing gradient gel electrophoresis of 16S rRNA gene fragments amplified from total community DNA to get insights into the effects of the inoculants and *R. solani* on the indigenous rhizosphere bacterial communities. Both inoculants showed a good colonization ability of the rhizosphere of lettuce with more than 10^6^ colony forming units per g root dry mass two weeks after planting. An effect of the soil type on rhizosphere competence was observed for 3Re4-18 but not for RU47. In both experiments a comparable rhizosphere competence was observed and in the presence of the inoculants disease symptoms were either significantly reduced, or at least a non-significant trend was shown. Disease severity was highest in diluvial sand followed by alluvial loam and loess loam suggesting that the soil types differed in their conduciveness for bottom rot disease. Compared to effect of the soil type of the rhizosphere bacterial communities, the effects of the pathogen and the inoculants were less pronounced. The soil types had a surprisingly low influence on rhizosphere competence and biocontrol activity while they significantly affected the bottom rot disease severity.

## Introduction

Plant pathogens are a limiting factor in crop productivity worldwide and responsible for yield losses [1]. Crop rotation, use of resistant cultivars and application of chemicals are strategies to minimize disease incidence and severity in Integrated Pest Management (IPM). However, resistant cultivars and effective fungicides for the control of diseases caused by soil-borne pathogens such as *Rhizoctonia solani* (Kühn) are often not available. Moreover, adverse eco-toxicological effects of chemical fungicides urge the development of alternative strategies to combat fungal diseases on crops [2]–[4]. In terms of disease control, it is well-documented that microbial inoculants as part of IPM can contribute to the reduction of adverse environmental effects caused by the exclusive reliance on fungicides [5]-[7] and thus represent a promising strategy for more sustainable agriculture [8]. Currently, the worldwide bio-pesticide market offers products including 60 bacterial and 60 fungal species [9]. Nonetheless, the exploitation of microbial inoculants as biocontrol agents in agriculture has been hampered by inconsistent results at the field scale [10], [11]. The inconsistency observed in biocontrol effects limits the attraction of microbial inoculants for growers but the reasons for this variability remain largely unexplored. Variation in the colonization ability of bacterial inoculants in the rhizosphere (rhizosphere competence) is assumed to be one of the factors contributing to this inconsistency. Several studies showed that the expression of genes, responsible for the capability of a biocontrol strain to suppress a disease, is often regulated in a cell density dependent manner [12], [13]. Therefore, the ability of inoculants to colonize the rhizosphere at sufficiently high numbers for an extended period was identified as a prerequisite for their beneficial effect on plants [11], [14], [15]. Thanks to the application of advanced genomics and microscopy methods the understanding of factors contributing to the biocontrol activity of bacterial strains has clearly improved [11], [15]–[18], especially for *Pseudomonas* strains such as *P. fluorescens* CHA0 or Pf-5. The complex regulation of genes involved in biocontrol and plant-microbe interaction has been studied in more detail [19]–[23]. Although the mode of action differs from strain to strain, numerous studies supported the assumption that biocontrol activity most likely results from multi-factorial processes such as antibiosis, production of cell wall degrading enzymes, surfactants, volatile substances or siderophores, competition for nutrients and space and/or the enhancement of plant innate defense responses [24], [25]. Several properties of bacterial inoculants such as motility [26], attachment [27], growth [16], production of antifungal metabolites or siderophores [24] and uptake and catabolism of root exudates [17], [28] have been shown to be linked to rhizosphere competence.

However, knowledge of factors influencing rhizosphere competence of bacterial inoculants under field conditions is largely lacking. Only in a few studies efforts were made to quantify the inoculant densities in the rhizosphere of field-grown crops or to evaluate the influence of inoculants and/or pathogens on the indigenous rhizosphere microbial community [7], [29], [30]. In agriculture, crops are cultivated under various ecological conditions. Therefore, a better understanding of the complex relationships among inoculant, pathogen, plant, and ecological factors such as the soil type is a prerequisite for improved and reliable biocontrol effects. So the goal of the present study was to investigate the influence of soil types on the rhizosphere competence and biocontrol activity of bacterial inoculants and their effects on the indigenous soil bacterial community.

The strains *Pseudomonas jessenii* RU47 [31] and *Serratia plymuthica* 3Re4-18 [32], which revealed remarkably good control effects against bottom rot in previous experiments [7], [32], were selected for this study. The causal agent of bottom rot on lettuce, the soil-borne fungus *R. solani* AG1-IB whose genome was recently sequenced [33] was used as model pathogen and lettuce as model host plant. The experimental plot system with three soil types under the same cropping history at the same field site enabled us to study the effects of different soil types on the rhizosphere competence and the biocontrol activity of the bacterial inoculants for the first time. We hypothesized that the soil types influence the rhizosphere competence and the biocontrol activity of the inoculant strains applied. Furthermore, we hypothesized that both the inoculants and the presence of the pathogen (*R. solani* AG1-1B) also influences the structural diversity of microbial communities in the rhizosphere of lettuce, and that the extent of this effect depends on the soil type.

## Materials and Methods

### Bacterial inoculants

The bacterial inoculant *P. jessenii* RU47 was isolated from a disease-suppressive soil [31], [34] and the strain *S. plymuthica* 3Re4-18 originated from the endorhiza of potato [35]. To monitor the survival of inoculants in the rhizosphere spontaneous rifampicin resistant mutants were used [34]. Both strains were stored at −80°C in Luria-Bertani broth (Carl Roth GmbH & Co. KG, Karlsruhe, Germany) with 20% glycerol.

### Design of field experiments

To evaluate the effect of soil types on the rhizosphere competence and disease suppression of the bacterial inoculants *P. jessenii* RU47 and *S. plymuthica* 3Re4-18 without and with *R. solani* inoculation, two independent field experiments were carried out in a unique experimental plot system at the Leibniz Institute of Vegetable and Ornamental Crops (Großbeeren, Germany, 52° 33′ N, 13° 22′ E). The first experiment was performed in unit 5, with planting on 8 June, harvest on 18 July 2011, the second experiment in unit 6, with planting on 27 July and harvest on 5 September 2011. Each unit was comprised out of three blocks (one block per soil type). The three soil types were characterized as Arenic-Luvisol (diluvial sand, DS), Gleyic-Fluvisol (alluvial loam, AL) and Luvic-Phaeozem (loess loam, LL) [36], [37]. Each block consisted of 24 plots of 2 m×2 m in size and a depth of 75 cm. In unit 5, the following crops were cultivated from 2000 to 2010: pumpkin, nasturtium, nasturtium, phacelia, amaranth, wheat, pumpkin, nasturtium, wheat, broccoli, wheat, Teltow turnip and lettuce, and in unit 6 pumpkin, nasturtium, pumpkin, amaranth, wheat, wheat, pumpkin, nasturtium, wheat, wheat and lettuce.

Lettuce seeds (cv. Tizian, Syngenta, Bad Salzuflen, Germany) were sown in seedling trays filled with the respective soil type and incubated at 12°C for 48 h and then grown in the greenhouse at approximately 20/15°C (day/night). To maintain the substrate moisture all seedling trays were watered daily and fertilized weekly (0.2% Wuxal TOP N, Wilhelm Haug GmbH & Co. KG, Düsseldorf, Germany). Lettuce seedlings were transplanted at the 3–4-leaf stage (BBCH 14) in the experimental plot system. Plants were placed in a within-row and intra-row distance of 30 cm (36 plants per plot), and lettuce plantlets were overhead irrigated based on the computer program ‘BEREST’ [38]. The daily soil water content in the rooted soil layer using the water holding capacity of the soil under consideration of the plant growth stage and the potential evapotranspiration were the input variables for the irrigation program. Irrigation decisions were made on the basis of the calculated soil water content and the expected evapotranspiration and precipitation of the next five days. The soil temperature (reflectometer PT100b1/3 DIN, Messtechnik Geraberg GmbH, Martinroda, Germany) and the matric potential (CS616-L water content reflectometer, Campbell Scientific, North Logan, Utah, USA) were recorded by data logger (4MbSRAm data logger, Campbell Scientific). Both reflectometers determine an average value of a 20 cm top soil layer. The fertilizer was added to each plot based on a chemical analysis of soils before planting, done according to the certified protocols of Agricultural Tests and Research Institutions Association (VdLUFA, Germany). Each soil type was adjusted to the same amount of nitrogen (162 mg/100 g) by fertilization with Kalkamon (27% N, TDG mbh Lommatzsch, Germany). Lettuce was harvested six weeks after planting (6WAP, BBCH 49) in both experiments. The lettuce shoot dry mass (SDM) of each plant and the disease severity of bottom rot were scored at harvest. For assessment of SDM each lettuce head was cut in four portions and dried at 80°C until a constant dry mass was achieved. The disease severity was rated in four categories: 1–without bottom rot symptoms; 3–symptoms only on first lower leaves and small brown spots on the underside of leaf midribs; 5–brown spots on leaf midribs on lower and next upper leaf layer and 7–severe disease symptoms on upper leaf layers and beginning of head rot to total head rot according to Grosch et al. [39].

The following treatments of lettuce were investigated for each soil type: no treatment with inoculants without (control) and with *R. solani* (*Rs*) inoculation (control+*Rs*), plants treated with inoculants without (RU47; 3Re4-18) and with *R. solani* inoculation (RU47+*Rs*; 3Re4-18+*Rs*). Each treatment included four replicates with 36 plants per replicate.

### Preparation of pathogen inoculum and inoculation

The *R. solani* AG1-IB isolate 7/3 from the strain collection of the Leibniz Institute of Vegetable and Ornamental Crops (Großbeeren) was used in the present study. The inoculum was prepared as described by Schneider et al. [40] on barley kernels. To ensure a higher pathogen pressure the following inoculation procedure was applied: 36 lettuce plants were planted in each of the 24 plots as described above; after a cultivation time of three weeks they were evenly incorporated into the top soil (10 cm) by means of a rotary hoe, together with 40 g of barley kernels without or with *R. solani* infestation. The experiment started two weeks later assuming a decomposition of incorporated infested or non-infested lettuce plant material.

### Preparation of bacterial inocula and application mode

For seed treatment King’s B agar (Merck KGaA, Darmstadt, Germany) supplemented with rifampicin (75 µg/ml) were inoculated with the *P. jessenii* RU47 of *S. plymuthica* 3Re4-18 and incubated overnight at 29°C. The bacterial cells were harvested from the Petri dishes by resuspension in 15 ml sterile 0.3% NaCl and the concentration was adjusted in a spectrophotometer to a density of 10^8^ colony forming units (CFU)/ml. A total of 200 lettuce seeds (cv. Tizian) were coated with 500 µl of a bacterial cell suspension dripping on the seed during vortexing in a 50 ml Falcon tube.

To prepare the inoculum for the treatment of young plants the inoculant strains were grown in nutrient broth (NB II, SIFIN GmbH, Berlin, Germany) amended with rifampicin (75 µg/ml) on a rotary shaker (90 rpm) at 29°C. After a cultivation time of 16 h the overnight culture was centrifuged at 13,000 *g* for 5 min, the supernatant discarded and the pellet was resuspended in sterile 0.3% NaCl solution. The cell density was adjusted to 10^7^ CFU/ml or 10^8^ CFU/ml for the drenching before and after planting, respectively. Lettuce plants were treated by drenching with 20 ml bacterial cell suspension per plant at the 3-leaf stage one week before planting in the field. A second treatment of young plants with 30 ml bacterial cell suspension 10^8^ CFU/ml per plant was carried out at the 4-leaf stage two days after planting. The control plants were drenched with 20 ml or 30 ml of 0.3% NaCl solution, respectively, instead of bacterial suspension.

### Sampling and sample processing

Rhizosphere samples were collected two and five weeks after planting (2WAP and 5WAP; BBCH 19 and BBCH 49) the lettuce in the experimental plot system. For each treatment and sampling time the roots of three plants per replicate (plot) were combined as a composite sample and considered as one replicate; four replicates were used per treatment. Adhering soil was removed by washing the roots with sterile tap water before microbial cells were extracted as follows: the roots were cut into pieces of approximately 1 cm length and carefully mixed. Five gram of roots were placed in sterile Stomacher bags and treated by a Stomacher 400 Circulator (Seward Ltd, Worthing, UK) for 30 s at high speed after adding 15 ml of sterile 0.3% NaCl. The Stomacher blending step was repeated three times and followed by centrifugation steps as described by Schreiter et al. [37].

### Analysis of rhizosphere competence of the bacterial inoculants

The ability of *P. jessenii* RU47 and *S. plymuthica* 3Re4-18 to colonize the rhizosphere of lettuce grown in the three soil types was determined 2WAP and 5WAP. Aliquots of the rhizosphere microbial cell suspension resulting from the combined supernatants of three Stomacher blending steps were immediately processed to determine the inoculant CFU counts by plating serial dilutions onto King’s B agar supplemented with rifampicin (75 µg/ml) and cycloheximide (100 µg/ml) and incubated at 29°C for 48 h. The CFU counts were calculated per gram of root dry mass (RDM). For all soil types Stomacher supernatants obtained from the control plots were plated as well to determine the background of the rifampicin resistant indigenous bacteria.

### Data analysis

Data of SDM, disease severity and inoculant plate counts were analyzed with the STATISTICA program (StatSoft Inc., Tulsa, OK, USA). The impact of the soil type, the pathogen and the inoculants on SDM was determined using three-way ANOVA (*P<*0.1) combined with Tukey post-hoc test (*P<*0.1). The data of disease severity was evaluated using the nonparametric Kruskal Wallis test followed by Mann-Whitney U-test (*P<*0.1). The determined inoculants density (CFU counts/g RDM) was calculated and logarithmically (Log_10_) converted before the impact of the soil type, plant growth development stage, and the presence of the pathogen *R. solani* on the plate counts of each inoculant strain was analyzed using three-way ANOVA (*P<*0.05) combined with Tukey post-hoc test (*P<*0.05). Average values for soil temperature of each soil type were analyzed by Tukey post-hoc test using standard errors of difference values and calculation of variance (*P<*0.05).

### Analysis of 16S rRNA gene fragments PCR amplified from total community DNA by denaturing gradient gel electrophoresis (DGGE)

Total community DNA (TC-DNA) was extracted from the microbial pellets using the FastDNA SPIN Kit (MP Biomedicals, Heidelberg, Germany) as described by the manufacturer after a harsh lysis step with the FastPrep-24 Instrument (MP Biomedicals, Heidelberg, Germany). The TC-DNA was purified with GENECLEAN SPIN Kit (MP Biomedicals, Heidelberg, Germany) according to the manufacturer. The purified TC-DNA was diluted 1∶10 with 10 mM Tris HCl before use.

For amplification of 16S rRNA gene fragments, PCR reactions were performed with TC-DNA obtained from rhizosphere samples with the primers F984-GC and R1378 as described by Heuer [41] using Taq DNA polymerase (Stoffel fragment, ABI, Darmstadt, Germany). The PCR products were analyzed by DGGE approach as described by Weinert et al. [42].

Bacterial fingerprints were evaluated with GELCOMPAR II version 6.5 (Applied Maths, Sint-Martens-Latern, Belgium) as described by Schreiter et al. [37]. The obtained Pearson similarity matrices were used for construction of a dendrogram by an Unweighted Pair-Group Method with Arithmetic mean (UPGMA) as well as of statistical analysis by the permutation test, calculating the d-value from the average overall correlation coefficients within the groups minus the average overall correlation coefficients between samples from treatments compared as suggested by Kropf et al. [43].

## Results

### Soil parameters of both field experiments

The concentration of N, P and K measured for each soil type before planting revealed only minor variations between both experiments (Table S1). In the second experiment, the temperature measured for all three soils in the 20 cm top layer was approximately 0.6°C below that of the first experiment (Table S2).

In contrast to the temperature the volumetric soil water content (VWC) in the top soils varied significantly among the soil types and experiments (except for AL and LL soil in the first experiment; Table S3). The lowest percentage of VWC was recorded in DS, and the highest in AL soil in both experiments. The calculated percentages of VWC in the first experiment were 18.9%, 39.6% and 39.1% in DS, AL and LL soil, and 17.0%, 35.7% and 27.4% in DS, AL and LL soil, in the second experiment. In the first experiment the VWC of AL and LL soil did not differ significantly. In both experiments VWC values for DS soil were significantly lower than those for AL and LL (Tukey post-hoc tests and confidence limits in Table S3).

### Effect of the soil types on rhizosphere competence of the bacterial inoculants

**Figure 1:**
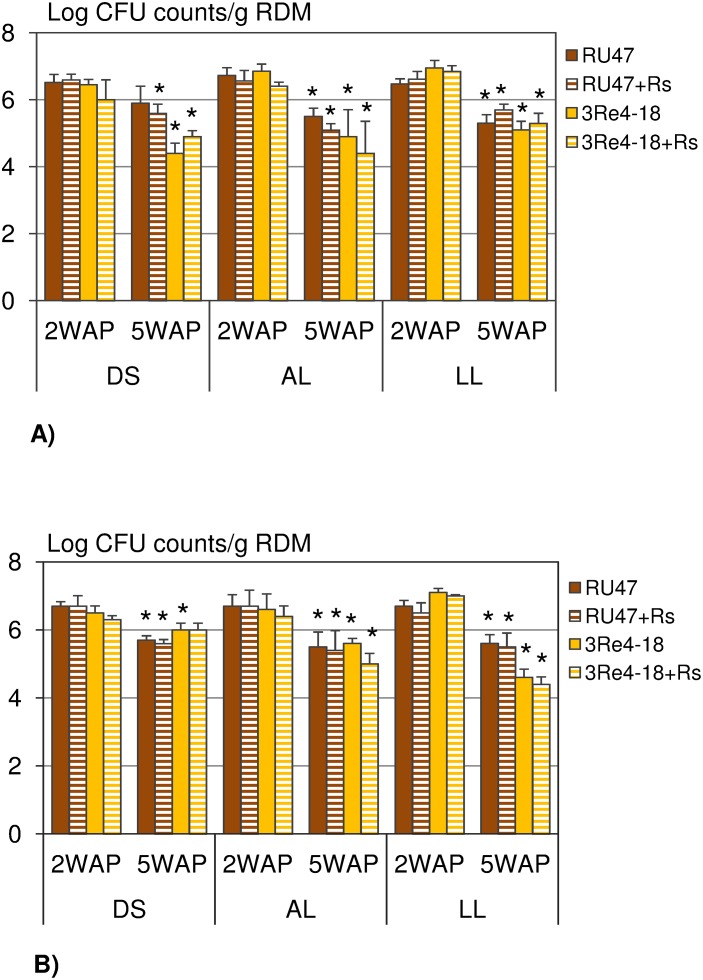
CFU counts of *Pseudomonas jessenii* RU47 and *Serratia plymuthica* 3Re4-18 per gram of root dry mass (RDM) without (RU47, 3Re4-18) and with *Rhizoctonia solani* inoculation (RU47+*Rs;* 3Re4-18+*Rs*) in two experiments, A) and B), two and five weeks after planting (2WAP, 5WAP) in the 2011-season. Plants were grown in three soil types (DS, AL, LL) at the same field site. An asterisk indicates significant differences in CFU counts of RU47 or 3Re4-18 between 2WAP and 5WAP for each soil type (Tukey post-hoc test, *P*<0.05). The bars show the standard deviation.

In both experiments the inoculants were able to colonize the rhizosphere of lettuce grown in the different soil types at comparable CFU counts (Figure 1). Three-way ANOVA (soil type, plant growth development stage, pathogen) revealed that in both experiments the soil type had no significant effect on the CFU of RU47 in the rhizosphere of lettuce (*P*>0.05) but on the CFU of 3Re4-18 (*P*<0.05) (Table 1). In the first and second experiment 2WAP the CFU counts of 3Re4-18 were significantly higher in LL soil than in DS and AL soil. With increasing plant age the CFU counts of both inoculants decreased in the rhizosphere of lettuce grown in all three soils and in both experiments (*P*<0.05) except for the treatment with RU47 in DS soil in the first experiment and the treatment with 3Re4-18+*Rs* in DS soil in the second experiment (Figure 1). No significant effect of *R. solani* on CFU counts of both inoculants was revealed (*P>*0.05) in both experiments. The natural background of the rifampicin mutation was very low in all soil types.

**Table 1:** ANOVA results: Factor [soil type, plant growth development stage (PGDS), pathogen] dependent *P*-values for CFU counts of *Pseudomonas jessenii* RU47 and *Serratia plymuthica* 3Re4-18 (*P*<0.05).

	Experiment 1		Experiment 2	
Factor	RU47	3Re4-18	RU47	3Re4-18
Soil type	0.079	0.0007	0.544	<0.0001
PGDS	<0.0001	<0.0001	<0.0001	<0.0001
Pathogen	0.623	0.2609	0.3872	0.006

### Effects of the soil type, the bacterial inoculants and the pathogen on the lettuce growth

**Figure 2:**
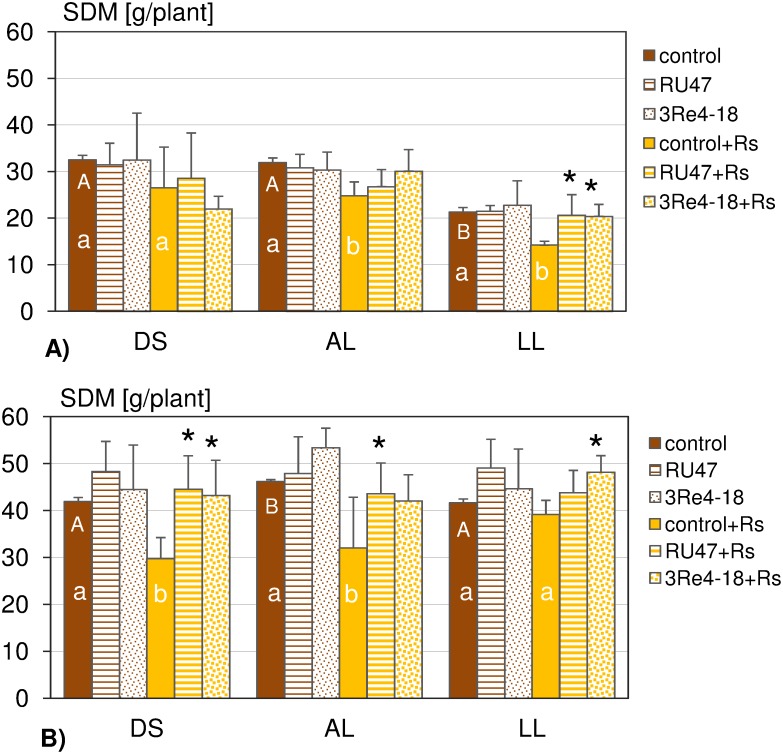
Shoot dry mass (SDM) of lettuce (cv. Tizian) determined for the following treatments: control, RU47, 3Re4-18, control+*Rs*, RU47+*Rs*, 3Re4-18+*Rs*) of two experiments, A) and B), in the 2011-season. Plants were grown in three soil types (DS, AL, LL) for six weeks each, at the same field site. Different capital letters denote significant differences in SDM of lettuce in controls or control+*Rs* between soil types (ANOVA; *P*<0.1). Different lower-case letters indicate significant differences in SDM between control and control+*Rs* within each soil type (Tukey post-hoc test, *P*<0.1). An asterisk denotes significant effects of the inoculants RU47 and 3Re4-18 on SDM to the corresponding control or control+*Rs* in each soil type. The bars show the standard deviation.

The effects of the soil type, the inoculants and *R. solani* on lettuce growth assessed by comparing the SDM of lettuce plants harvested 6WAP revealed lower lettuce growth in the first experiment compared with the second experiment in particular for LL soil (Figure 2). A significant effect of the soil type on lettuce growth was observed in both experiments. However, the results differed between the experiments. In the first experiment plants grown in LL soil showed the lowest SDM (21.3 g/plant) compared to SDM of plants from DS (32.5 g/plant) and AL soil (31.9 g/plant). In the second experiment the SDM was highest for plants grown in AL soil (46.2 g/plant) while the SDM of plants from DS (41.9 g/plant) and LL soil (41.6 g/plant) were comparable. In contrast to the first experiment an improved lettuce growth was observed in the treatments with the inoculants RU47 and 3Re4-18 in all three soils in the second experiment. However, the inoculant effects on lettuce growth were not significant (Figure 2).

In general the pathogen *R. solani* had a negative effect on lettuce growth in all three soils and in both experiments. The effect was significant in AL soil in both experiments, in LL soil only in the first experiment, and in DS soil in the second experiment (Figure 2). The treatment of lettuce with the inoculants resulted in an improved lettuce growth in RU47+*Rs* and 3Re4-18+*Rs* in all three soils and in both experiments but significant effects were only recorded in the first experiment for the treatments RU47+*Rs* in DS soil and 3Re4-18+*Rs* in LL soil, and in the second experiment for RU47+*Rs* and 3Re4-18+*Rs* in DS soil, for RU47+*Rs* in AL soil and for 3Re4-18+*Rs* in LL soil (Figure 2).

### Effect of the soil type on the biocontrol activity of the bacterial inoculants

**Figure 3:**
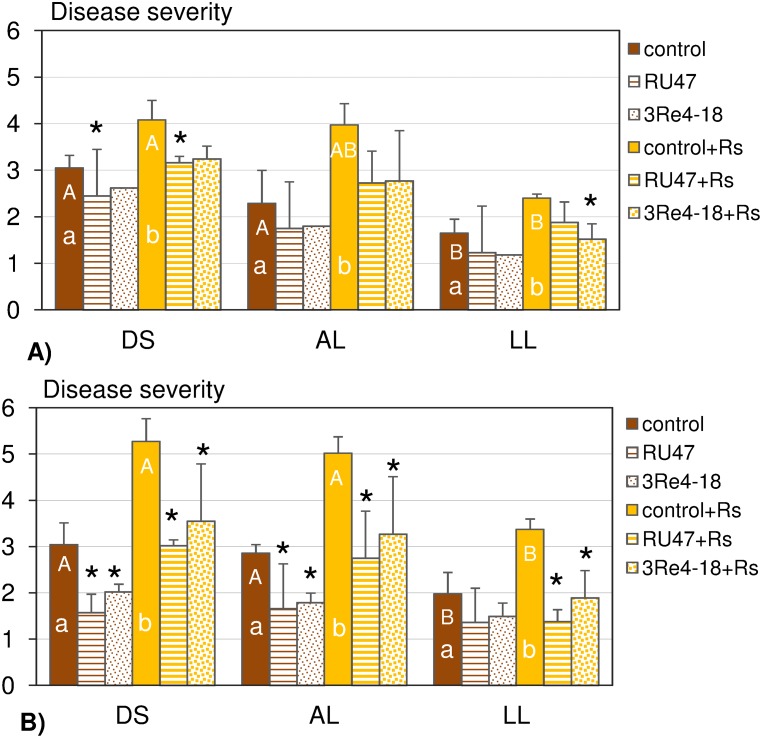
Disease severity of bottom rot on lettuce (cv. Tizian) determined for the following treatments: control, RU47, 3Re4-18, control+*Rs,* RU47+*Rs* and 3Re4-18+*Rs* the in two experiments, A) and B) in the 2011-season. Plants were grown in three soil types (DS, AL, LL) for six weeks each, at the same field site. Different capital letters indicate significant differences in disease severity of bottom rot in controls or control+*Rs* between soil types and different lower-case letters indicate significant differences between control and control+*Rs* within each soil type (Mann-Whitney U-test; *P*<0.1). An asterisk denotes significant differences in disease severity of the RU47 or 3Re4-18 treatment compared to the control and of RU47+*Rs* or 3Re4-18+*Rs* treatments compared to the control+*Rs* in each soil type. The bars show the standard deviation.

Bottom rot symptoms were also recorded in the untreated controls in each soil and in both experiments (Figure 3). The inoculation of *R. solani* (control+*Rs*) resulted in a significantly increased disease severity in all three soils and in both experiments. More severe bottom rot symptoms were scored in the second compared to the first experiment based on the disease severity in the pathogen controls in all three soils. The soil type influenced the disease severity of bottom rot in both experiments (Figure 3). The lowest disease severity was always recorded on lettuce grown in LL soil (control and control+*Rs*) compared to disease severity observed for lettuce grown in DS and AL soil.

RU47 and 3Re4-18 were able to reduce severity of bottom rot on lettuce in the treatments without and with *R. solani* inoculation in all three soils and in both experiments (Figure 3). Significant biocontrol effects were observed by the inoculant RU47 in DS soil (RU47; RU47+*Rs*) in both experiments and in AL (RU47; RU47+*Rs*) and LL (RU47+*Rs*) soil in the second experiment only. The inoculant 3Re4-18 showed a significant control effect in LL (3Re4-18+*Rs*) in the first experiment and in all soils in the second experiment (3Re4-18; 3Re4-18+*Rs*) except for 3Re4-18 in LL soil (Figure 3).

### Effects of the pathogen and the bacterial inoculants on the bacterial community composition in the rhizosphere of lettuce

The effect of *R. solani* on the bacterial community composition were assessed by comparing the bacterial DGGE fingerprints of the control treatments (control) with those of the pathogen control (control+*Rs*) for each of the soils. In both experiments, the inoculation of *R. solani* had low but an significant effect in all soils except LL in the second experiment, the highest d-values were observed in DS soil (Table 2).

**Table 2:** Treatment-dependent differences (d-values) of bacterial communities in the rhizosphere of lettuce, grown in three soil types (DS, AL, LL), in the 2011-season.

			Differences between control and				
Soil type	Experiment	Figure	control+*Rs*	RU47	3Re4-18	RU47+*Rs*	3Re4-18+*Rs*
DS	1	S1	6.1*	2.6*	6.2*	3.6*	10.3*
AL	1	S2	1.9*	13.6*	20.6*	12.3*	13.2*
LL	1	S3	1.7*	6.8*	10.1*	7.8*	10.8*
DS	2	S4	4.9*	4.4*	8.8*	5.7*	7.6*
AL	2	4a	2.9*	2.8	10.0*	9.0*	17.3*
LL	2	S5	3.5	14.6*	16.1*	16.2*	21.8*

The asterisks indicate the significant differences (*P*<0.05) between the untreated control and the respective treatment.

**Figure 4:**
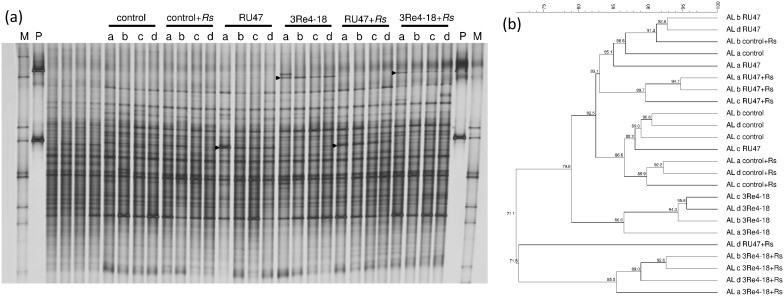
DGGE fingerprints (a) of bacterial 16Sr-RNA gene fragments from community DNA extracts were obtained from lettuce rhizosphere 2WAP of lettuce into the AL soil (second experiment). The corresponding UPGMA dendrogram (b) of these DGGE fingerprints based on the Pearson similarity matrix. The four replicates of the treatments: control, control+*Rs,* RU47, 3Re4-18, RU47+*Rs,* 3Re4-18*+Rs* were indicated by a–d. M: marker [57].

The effects of the inoculants on the bacterial community composition in the rhizosphere of lettuce analyzed by DGGE varied between the two experiments. The bacterial 16S rRNA gene fragments, amplified from TC-DNA of all treatments, were analyzed for each soil type separately and the DGGE gel for AL soil of the second experiment that had high d-values (Figure 4a). The DGGE fingerprint (Figure 4a) revealed that 2WAP the inoculant strain 3Re4-18 belonged to the dominant members of the bacterial community in the rhizosphere as bands with the electrophoretic mobility of 3Re4-18 were detected in the corresponding treatments. However, bands with electrophoretic mobility of the inoculant RU47 could not be detected as it co-migrated with bands of the indigenous bacterial community. Thus RU47 could only be identified in the second experiment in DS and AL soil, while 3Re4-18 was detected in AL and LL soil in the first, and in DS and AL soils in the second experiment (Figures S1–S5). Furthermore, the analysis of DGGE fingerprints by UPGMA revealed that the bacterial community composition of the different treatments shared approximately 70% similarity for all soils (Figure 4b). Although treatment dependent clusters were not always observed, the treatment effects were significant based on the permutation test (*P*<0.05) for all treatments with the inoculants RU47 or 3Re4-18 except for RU47 in AL soil in the second field experiment. However, the differences between the fingerprints of the controls and the treatments given by the calculated d-values varied for both experiments (Table 2). In the first experiment highest d-values were observed for AL soil for both inoculants while in the second experiment the highest d-values were observed for LL soil (Table 2). In both experiments and in all soils higher d-values for the treatments with 3Re4-18 indicated a stronger effect of this inoculant on the indigenous rhizosphere bacterial community compared to RU47 (Table 2). The effects of both inoculants on the bacterial community composition were typically increased for the treatments with *R. solani* inoculation in both experiments (except for RU47 and 3Re4-18 in AL soil in the first experiment).

## Discussion

The availability of three soil types differing in their properties but sharing the same cropping history and weather conditions at the same field site enabled us to investigate the effect of the soil type on the rhizosphere competence of the two bacterial inoculants *P. jessenii* RU47 and *S. plymuthica* 3Re4-18 without and with *R. solani* inoculation for the first time. We hypothesized that the soil types with their distinct microbial community composition and physico-chemical properties but also the soil type dependent root exudates of the model plant lettuce [44] might affect the rhizosphere competence of the inoculants and in consequence their biocontrol efficacy. The composition of root exudates collected from lettuce grown in DS, AL and LL soil was recently shown to differ only quantitatively as similar compounds were detected as independent of the soil type [44]. Several studies showed that root exudates are important drivers of the rhizosphere microbial community composition [45], [46] and the metabolic activity of the bacterial community including inoculant strains [47]. Recently, we could show by means of DGGE and pyrosequence analysis of 16S rRNA gene fragments, amplified from TC-DNA of bulk soil samples taken from the experimental unit 6 in 2010, that the three soil types indeed harbored a distinct bacterial community structure indicating the importance of the mineral composition and the soil organic matter for shaping the bacterial community composition [37]. Interestingly, in the rhizosphere of lettuce grown in the different soils numerous similar genera were increased in relative abundance, and a high proportion of dominant operational taxonomic units were shared among the rhizosphere samples from different soils [37]. Among the genera, significantly increased in the rhizosphere, was the genus *Pseudomonas* which might explain the negligible effect of soil types on the ability of RU47 to colonize the lettuce rhizosphere in both experiments performed in 2011. Remarkably, similar CFU counts of RU47 were observed 2WAP in all soil types and in both experiments. The decrease of RU47 CFU counts in the rhizosphere observed 5WAP in both experiments might well be a decrease in its relative abundance per gram of RDM as the rhizosphere pellet was obtained from the complete root system due to changes in the rhizoplane surface/RDM ratio or/and possibly newly emerging roots were not colonized by RU47 as previously reported for *gfp*-tagged inoculants [48]. A decrease in inoculant CFU densities with increasing plant age of lettuce confirmed previous observations for the inoculants used in this study [34], [49] and other biocontrol strains [24]. Nonetheless, our results showed an influence of the soil types on the rhizosphere competence of 3Re4-18. While higher CFU counts were observed in LL soil in both experiments 2WAP in comparison to DS and AL soil, a pronounced decline of 3Re4-18 counts was recorded in LL soil in the second experiment 5WAP. Indeed, in the second experiment the SDM was almost doubled for lettuce grown in LL soil compared to the first experiment which might have resulted in a decreased relative abundance of 3Re4-18. Neumann et al. [44] reported that the lettuce growth and the root morphology were influenced by soil type which might have contributed to the soil type dependent differences in rhizosphere competence of 3Re4-18. The differences in lettuce growth between the first and the second experiment in particular in LL soil were not unusual for agricultural practice. Factors that might have contributed to these differences range from slightly lower VWC, temperatures in the field directly after planting to differences in sun light intensity. In addition, the slightly different cropping history in both experimental units could have affected the soil microbiome with potential effects on plant growth. Plant growth in turn is assumed to influence the quantity of root exudates [50]. Several studies have underlined a clear relationship between the level of disease suppression and inoculant densities in the rhizosphere of an introduced biocontrol strain [17], [51], [52]. Inconsistent results in biological control were often assumed to be associated with inefficient root colonization at the field scale [11], [53]. However, despite the good colonization of the lettuce rhizosphere by both inoculants in the present study, the biocontrol effects varied in both experiments. Although in the presence of the inoculant strains the disease severity was reduced. Compared to the control and the pathogen control (control+*Rs*), the biocontrol effects were not always significant. In fact, in the first experiment significant biocontrol effects were only observed for RU47 and RU47+*Rs* in DS soil and 3Re4-18+*Rs* in LL soil while biocontrol effects were found to be much more pronounced and significant in almost all treatments in the second experiment. The improved biocontrol effect seems to be correlated with an increased lettuce growth in the second experiment. It is tempting to speculate that an improved plant growth might be linked to an increased amount of photosynthates released via the roots. The increased root exudation in turn might have not only affected the metabolic activity of the inoculants but also facilitated the interaction of *R. solani* with the lettuce plant. Therefore, more severe bottom rot symptoms in the control and the control+*Rs* treatments were recorded in the second experiment. Furthermore, the disease severity in the control (natural background of *R. solani*) and in the pathogen control (control+*Rs*) was highest in DS soil and lowest in LL soil in both experiments. The higher conduciveness of the DS soil may be caused by higher oxygen availability because of the bigger pore sizes in sandy soils but might also be due to differences in the microbial community structure. Also, Steinberg et al. [54] reported that the capacity of a soil to antagonize the action of a plant pathogen is related to the structure and function of the microbial communities in soil.

Molecular fingerprinting techniques confirmed that the inoculants were indeed dominant members of the bacterial communities in the rhizosphere of lettuce. The additional DGGE bands of the inoculants strains 3Re4-18 and the increased intensity of a band co-migrating with RU47 which did not occur in the control treatments likely contributed to the effects of the inoculants on the rhizosphere bacterial community composition determined by the permutation test (Table 2). An approach to prevent the amplification of the inoculant strains is the use of taxon-specific primers which would exclude the taxonomic group to which the inoculant belongs, as proposed by Gomes et al. [55]. The actinobacterial fingerprint, done for the samples from 2WAP of the second experiment still revealed a significant effect except for RU47 in DS soil (Schreiter et al. unpublished data). The low effects of *R. solani* on the bacterial community composition can most likely be explained by the pathogenesis of *R. solani* AG1-IB which is primarily not a root pathogen but attacks the lower leaves of lettuce [56]. In conclusion, in the present study which was based on two independent field experiments performed in the 2011-season we could not confirm our hypothesis that the soil type influences the rhizosphere competence of biocontrol strains RU47 and 3Re4-18. We are aware that this finding cannot be generalized and can be different for other plants–inoculant combinations. The soil type independent enrichment of *Gammaproteobacteria* such as *Pseudomonas,* as observed by Schreiter et al. [37], in the rhizosphere of lettuce may be an explanation for the fact that the rhizosphere competence of RU47 was not influenced by soil type. Only minor changes in the bacterial rhizosphere composition were observed due to the inoculation of RU47, 3Re4-18 and *R. solani*, and these did not depend on the soil type. However, the plant growth and the disease severity of bottom rot were influenced by the soil type which in turn also influenced the biocontrol effects. The present study is unique as the rhizosphere competence, biocontrol effects, plant growth and treatment effects on the indigenous rhizosphere bacterial community were assessed in three soil types at the same site in two independent field experiments. The present study showed that the often reported inconsistency of biocontrol is likely more due to plants which in turn are influenced by weather conditions. Thus we conclude that the multitrophic interaction between plant, inoculant, pathogen and indigenous microbial community deserves far more attention in the future.

## Supporting Information

Figure S1 ***Bacteria*** **DGGE fingerprint of rhizosphere-samples were obtained from DS soil 2WAP of the lettuce (first experiment).** Lanes a–d replicates of each of the treatments: untreated control (control), control inoculated with *Rhizoctonia solani* (control+*Rs*), inoculation with *Pseudomonas jessenii* RU47 (RU47), inoculation with *Serratia plymuthica* 3Re4-18 (3Re4-18), inoculation with RU47 and *R. solani* (RU47+*Rs*), inoculation with 3Re4-18 and *R. solani* (3Re4-18*+Rs*); M: marker [57].
(TIF)

Figure S2 ***Bacteria*** **DGGE fingerprint of rhizosphere-samples** **were obtained from AL soil 2WAP of the lettuce (first experiment).** Lanes a–d replicates of each of the treatments: untreated control (control), control inoculated with *Rhizoctonia solani* (control+*Rs*), inoculation with *Pseudomonas jessenii* RU47 (RU47), inoculation with *Serratia plymuthica* 3Re4-18 (3Re4-18), inoculation with RU47 and *R. solani* (RU47+*Rs*), inoculation with 3Re4-18 and *R. solani* (3Re4-18*+Rs*); P: inoculants (upper band 3Re4-18; lower band RU47); M: marker [57].
(TIF)

Figure S3 ***Bacteria*** **DGGE fingerprint of rhizosphere-samples were obtained from LL soil 2WAP of the lettuce (first experiment).** Lanes a–d replicates of each of the treatments: untreated control (control), control inoculated with *Rhizoctonia solani* (control+*Rs*), inoculation with *Pseudomonas jessenii* RU47 (RU47), inoculation with *Serratia plymuthica* 3Re4-18 (3Re4-18), inoculation with RU47 and *R. solani* (RU47+*Rs*), inoculation with 3Re4-18 and *R. solani* (3Re4-18*+Rs*); P: inoculants (upper band 3Re4-18; lower band RU47); M: marker [57].
(TIF)

Figure S4 ***Bacteria*** **DGGE fingerprint of rhizosphere-samples were obtained from DS soil 2WAP of the lettuce (second experiment).** Lanes a–d replicates of each of the treatments: untreated control (control), control inoculated with *Rhizoctonia solani* (control+*Rs*), inoculation with *Pseudomonas jessenii* RU47 (RU47), inoculation with *Serratia plymuthica* 3Re4-18 (3Re4-18), inoculation with RU47 and *R. solani* (RU47+*Rs*), inoculation with 3Re4-18 and *R. solani* (3Re4-18*+Rs*); P: inoculants (upper band 3Re4-18; lower band RU47); M: marker [57].
(TIF)

Figure S5 ***Bacteria*** **DGGE fingerprint of rhizosphere-samples were obtained from LL soil 2WAP of the lettuce (second experiment).** Lanes a–d replicates of each of the treatments: untreated control (control), control inoculated with *Rhizoctonia solani* (control+*Rs*), inoculation with *Pseudomonas jessenii* RU47 (RU47), inoculation with *Serratia plymuthica* 3Re4-18 (3Re4-18), inoculation with RU47 and *R. solani* (RU47+*Rs*), inoculation with 3Re4-18 and *R. solani* (3Re4-18*+Rs*); P: inoculants (upper band 3Re4-18; lower band RU47); M: marker [57].
(TIF)

Table S1 **Concentration of nitrogen (N), phosphorus (P) and potassium (K) in soil measured before planting lettuce in three soil types (diluvial sand, DS; alluvial loam, AL; loess loam LL) in the season 2011 of the experimental plot system at the same field site.**
(DOCX)

Table S2 **Soil temperature recorded on average of the upper 20 cm top soil in three soil types (diluvial sand, DS; alluvial loam, AL; loess loam, LL) in the season 2011 at the same field site.**
(DOCX)

Table S3 **Comparison of the volumetric soil water content (VWC) of three soil types (diluvial sand, DS; alluvial loam, AL; loess loam, LL) in the season 2011 at the same field site.**
(DOCX)
