# Supplementary material for: Soil Type Dependent Rhizosphere Competence and Biocontrol of Two Bacterial Inoculant Strains and Their Effects on the Rhizosphere Microbial Community of Field-Grown Lettuce
Source: PLoS One. 2014 Aug 6;9(8):e103726. doi: 10.1371/journal.pone.0103726 (PMC4123886; doi:10.1371/journal.pone.0103726)
Supplement: Table S1 — Concentration of nitrogen (N), phosphorus (P) and potassium (K) in soil measured before planting lettuce in three soil types (diluvial sand, DS; alluvial loam, AL; loess loam LL) in the season 2011 of the experimental plot system at the same field site. (DOCX) [file pone.0103726.s006.docx]

**Table S1.** Concentration of nitrogen (N), phosphorus (P) and potassium (K) in soil measured before planting lettuce in three soil types (diluvial sand, DS; alluvial loam, AL; loess loam LL) in the season 2011 of the experimental plot system at the same field site.

|  |  | **Experiment 1** | | |  |  | | **Experiment 2** | | | | |  |  |
| --- | --- | --- | --- | --- | --- | --- | --- | --- | --- | --- | --- | --- | --- | --- |
| **Soil type** | **pH** | **N*** | **P*** | **K *** | | | **N*** | | **P*** | | **K*** | | | |
| DS | 6.1 | 70.5 | 23.0 | 11.0 | | | 78.0 | | | 24.0 | | 12.0 | | |
| AL | 6.6 | 156.0 | 47.0 | 27.0 | | | 159.0 | | | 47.0 | | 26.0 | | |
| LL | 7.3 | 162.0 | 27.0 | 19.0 | | | 157.0 | | | 27.0 | | 23.0 | | |

*N, P, K content before fertilizer application [mg/100 g]

The concentration of N, P, K was analyzed according to the certified protocols of Agricultural Tests and Research Institutions Association (VdLUFA, Germany).
