# Supplementary material for: Soil Type Dependent Rhizosphere Competence and Biocontrol of Two Bacterial Inoculant Strains and Their Effects on the Rhizosphere Microbial Community of Field-Grown Lettuce
Source: PLoS One. 2014 Aug 6;9(8):e103726. doi: 10.1371/journal.pone.0103726 (PMC4123886; doi:10.1371/journal.pone.0103726)
Supplement: Table S2 — Soil temperature recorded on average of the upper 20 cm top soil in three soil types (diluvial sand, DS; alluvial loam, AL; loess loam, LL) in the season 2011 at the same field site. (DOCX) [file pone.0103726.s007.docx]

**Table S2.** Soil temperature recorded on average of the upper 20 cm top soil in three soil types (diluvial sand, DS; alluvial loam, AL; loess loam, LL) in the season 2011 at the same field site.

| **Experiment** | **Growth** | **Temperature** |  | **Soil type** | | |  |
| --- | --- | --- | --- | --- | --- | --- | --- |
|  | **Period** | **[°C]** | **DS** | | **AL** | **LL** | |
|  | 08. June to | on average | 19.0 | | 19.1 | 18.8 | |
| **1** | 19. July | min | 12.9 | | 13.9 | 12.9 | |
|  |  | max | 28.6 | | 27.7 | 27.1 | |
|  | 27. July to | on average | 18.3 | | 18.5 | 18.3 | |
| **2** | 07. September | min | 11.5 | | 12.5 | 12.1 | |
|  |  | max | 28.3 | | 27.1 | 26.5 | |

No significant differences in soil temperature between the soil types were revealed according to Tukey post-hoc test (*P*> 0.05).
