# Supplementary material for: Soil Type Dependent Rhizosphere Competence and Biocontrol of Two Bacterial Inoculant Strains and Their Effects on the Rhizosphere Microbial Community of Field-Grown Lettuce
Source: PLoS One. 2014 Aug 6;9(8):e103726. doi: 10.1371/journal.pone.0103726 (PMC4123886; doi:10.1371/journal.pone.0103726)
Supplement: Table S3 — Comparison of the volumetric soil water content (VWC) of three soil types (diluvial sand, DS; alluvial loam, AL; loess loam, LL) in the season 2011 at the same field site. (DOCX) [file pone.0103726.s008.docx]

**Table S3.** Comparison of the volumetric soil water content (VWC) of three soil types (diluvial sand, DS; alluvial loam, AL; loess loam, LL) in the season 2011 at the same field site.

| **Experiment** | **Soil types** | **estimate** | **SE** | **DF** | **P** | **CIl** | **CIu** |
| --- | --- | --- | --- | --- | --- | --- | --- |
| **1** | AL-DS | 0.2070 | 0.004854 | 121.0 | <0.0001 | 0.1955 | 0.2185 |
| **1** | AL-LL | 0.0045 | 0.004914 | 121.0 | 0.6348 | -0.0072 | 0.0161 |
| **1** | DS-LL | -0.2025 | 0.004914 | 121.0 | <0.0001 | -0.2142 | -0.1909 |
| **2** | AL-DS | 0.1864 | 0.003256 | 65.4 | <0.0001 | 0.1786 | 0.1942 |
| **2** | AL-LL | 0.0823 | 0.003915 | 81.9 | <0.0001 | 0.0729 | 0.0916 |
| **2** | DS-LL | -0.1041 | 0.003162 | 67.0 | <0.0001 | -0.1117 | -0.0966 |
| **1-2** | AL | 0.0396 | 0.004830 | 74.6 | <0.0001 | 0.0299 | 0.0492 |
| **1-2** | LL | 0.1173 | 0.004030 | 80.0 | <0.0001 | 0.1093 | 0.1254 |
| **1-2** | DS | 0.0189 | 0.003740 | 59.0 | <0.0001 | 0.0114 | 0.0264 |

For measuring the VWC ($\theta$, 0-20 cm soil depth) we used four time-domain reflectometers (TDR) CS625 (Campbell Scientific Inc., 2011) per soil. The necessary temperature correction of TDR data was done via $\tau_{c}=\tau_{u}+\left( 20-T_{soil} \right)\times\left( 0.526-0.052\tau_{u}+0.00136\tau_{u}^{2} \right)$ where $\tau_{c}$ and $\tau_{u}$ [$\mu s$] were corrected and uncorrected output and $T_{soil}$ [$^{\circ}C$] was soil temperature and then $\theta$ [$cm^{3}/cm^{3}$] was computed by $\theta=-0.0663-0.0063\tau_{c}+0.0007\tau_{c}^{2}$ (Campbell Scientific Inc., 2011). Afterwards $\theta$ was corrected using additional measurements of simultaneously determined TDR and gravimetric water content (GWC, reference method) measurements (data not shown). A linear regression analysis between GWC and TDR yielded the calibration function $\theta_{c}=0.64761\theta_{u} + 0.08993$ ($n=12$; $R_{adjusted}^{2}=0.8638$) where $\theta_{c}$ and $\theta_{u}$ were the corrected and uncorrected $\theta$, respectively. Comparisons of mean $\theta$ of soil types were done via ANOVA, assuming thereby homoscedasticity for experiment 1 heteroscedasticity for experiment 2. Corresponding *P*-values from null model likelihood ratio test were 0.1776 and 0.0012. For comparisons of the two experiments, we used t‑tests with preliminary F‑tests and found homoscedasticity for LL (*P*= 0.6545) and heteroscedasticity for AL (*P*= 0.0392) and DS (*P* <0.0001). All computations involving $\theta$ were performed with the DATA-step and the MEANS, MIXED, REG and TTEST procedures of SAS 9.2 (SAS Institute Inc. 2008).
